# Puerperal Convulsions

**Published:** 1877-01

**Authors:** 


Puerperal Convulsions.—*Read before the Philadelphia County Medical Society, By W. H. Parish, M.D., Obstetrician to the Philadelphia Hospital.*—Puerperal convulsions are, perhaps, almost as old as the puerperal process itself, yet so terrible are the eclamptic phenomena, so awful the consternation they produce on friends, or even on physicians, so frequently futile are the efforts of the profession, that the members of a medical body are always ready to listen to whatever pertains to this subject.

It is mainly with the view, then, of adding what I may to the mass of *data* which time collects that I present the histories of the following five cases. It is also with the view of eliciting an expression of opinion from the gentlemen present that I present these cases, and that I also subjoin some concise remarks bearing on what seem the most important relationships of the subject.

### Case 1.—Hysterical Convulsions During the Fifth Month of Pregnancy.

In August, 1872, I was called to a young woman, Sarah G., aged twenty years, unmarried, and in the contortions of a severe convulsion. The convulsion did not seem to partake of the character of an epileptiform seizure. The movements of the extremities did not seem wholly independent of volition. Following the convulsion was neither quiet stupor nor coma, but a state of apparent drowsiness, varied alternately with laughing and crying, and with ludicrous remarks. In a short time there was a repetition of the convulsion, and a number of them during the night. There were two convulsions during the following three days. She confessed to me that she was in the fifth month of an illegitimate pregnancy. During the following few weeks she had several convulsions, all of a hysterical character, but none during the later months of her pregnancy, and none during her lying in. Her urine was not albumenous, and there was no œdema. She never had a convulsion prior to the night to which I have referred.

### Case 2.—Epileptic Convulsions During Labor, and Recurring Subsequently.

On August 2d, 1874, I was in attendance on Mrs. R., in her fourth confinement. She was about thirty-five years old, married, and during her first two labors had experienced no convulsive manifestations. At her third labor, as the head was being born, a convulsion came on. During the lying-in, several convulsions occurred, and subsequently, at irregular periods, attacks having the characteristics of epileptic seizures would occur. During her fourth pregnancy the attacks continued, though with less freqnency than prior to the time of conception. As the fourth labor progressed, she manifested unusual agitation and alarm, but it was not until the placenta was being delivered that a convulsion occurred. Tonic followed by clonic spasms, unconsciousness, and a short coma, furnished the features of the seizures. In fifteen minutes a second convulsion occurred. Immediately after the first convulsion I gave her:

℞.—Morph. Sulph., . . . . . . . gr.⅓

Pot. Bromid., . . . . . . . gr.xxx.

After the second, I repeated the same remedies. During that lying-in there was no further return of the convulsions. At the time of her labor she was thin, delicate, with a troublesome cough, and with roughened breathing at the left apex. An examination of the urine passed after the convulsion showed no albumen. At differeut times since her getting up I have seen her in epileptic paroxysms.

Dr. R. A. Cleeman saw the patient with me.

Case 3.—Eclamptic Convulsions During Labor—Albumenuria.

In April, 1874, I was called to see a patient, then in labor at full term. She was an out-door patient of the Howard Hospital, and when I first saw her at her home, she was in active labor. She was about twenty five years of age, robust in appearance, of full habit; was, in short, a strong, red-faced Irish woman, and had passed through one previous labor without any untoward circumstance. There was not the slightest discernable œdema of any part. The pelvis was roomy, the head penetrating in the L. O. A. position; the os the size of a half dollar, and its borders not rigid. There was, from the moment that I saw her, one symptom, and only one, which, while it occasioned the patient absolute suffering, awakened no inconsiderable anxiety in myself. That one was an intense headache, referred mainly to the forehead, causing so much pain that she would clasp her hands to her head, and would cry, "my head, oh, my head," seeming unmindful of the pain that the vigorously contracting uterus must have occasioned. For a few days she had been passing her urine frequently, but in small quantities, and her bowels had been daily moved.

Apprehending that the head symptom was indicative of a condition that might be productive of a more serious phenomena, convulsions, I sent for chloral hydrate and ether. Before their arrival, however, there was an escape of the liquor amnii, and the uterus began to contract with even increased vigor, and the woman's great restlessness increased. A moment later, for an instant, she was still, then a slight drawing of the head and face to the left side, with twitchings of the muscles of the left half of the body, and then a fully-developed general convulsion, with complete loss of consciousness, flushed and livid features, dilated pupils, and a full, strong, bounding pulse. As quiet was returning, I made a hurried digital examination, and found the head ready to engage at the superior strait and the os amply dilated. Applying the forceps (Wallace's), I promptly delivered the woman, during the stage of coma, without injury to her tissues, and in a few minutes removed the placenta by Crede's method. The uterus contracted firmly and remained so, with the loss of not more than three or four ounces of blood. The coma following the convulsion lasted about ten minutes. The pulse lost its fullness and strength, and consciousness gradually returned. Thirty grains of chloral were now given by the mouth. But in a few minutes there was a recurrence of the convulsion, with all the attendant phenomena, save the coma was more prolonged, and the return to consciousness was not so complete, a state of great agitation and of resistance developing. During this and the subsequent convulsive seizure, I administered ether by inhalation. After the second convulsion, I gave thirty grains more of chloral, and before the third convulsion, Dr. W. B. Atkinson, Obstetrician to the Howard Hospital, arrived. He approved of the chloral and ether treatment, especially the chloral, this to be continued in doses of twenty grains every fifteen minutes until the convulsions ceased; the ether to be given only on the approach of the spasms. Within the first half hour subsequent to the first convulsion, one drachm of chloral hydrate had been given by the mouth, and yet a third convulsion occurred; a third one, too, more severe than the first, though held in partial check by the ether. Twenty grains more of chloral were given by mouth, and owing to the great difficulty now in making the patient swallow, forty grains were injected into the rectum. The convulsions simply recurred with increasing frequency, convulsion giving place only to coma, and coma soon only to convulsion; yet we continued the chloral *per rectum*, in drachm doses every half hour, until we had used up, in addition to the eighty grains by the mouth, 240 grains *per rectum*. Gradually the flushed and lived features gave place to pale and livid ones, and the pulse showed a heart losing its strength. At the expiration of the ninth hour the convulsions ceased. The nerve centres had become exhausted, because of the repeated abnormal explosion of nerve force; or they had become overpowered by reason of vascular engorgement, or of œdema, or of hemorrhagic exudation; or it is probable they were narcotized, because of uræmia, with or without additional toxæmia, or narcotized also, may it not have been, by reason of chloral?

After two hours of coma the patient died. The child was a female, of large size, and is still living. The patient's urine, passed after the beginning of labor, but before the beginning of the convulsions, showed, on testing, one-half in bulk to be albumen. There was no microscopical examination. After the appearance of the convulsive seizures there was almost complete suppression of urine. No autopsy could be obtained.

### Case 4.—Eclamptic Convulsions Subsequent to Labor—Albumenuria.—High Temperature.

Mary E. H., aged 22 years, a primipara, entered the Philadelphia Hospital several weeks prior to her confinement. She stated that she was married, but confessed later that her pregnancy was an illegitimate one. During the time she was in the hospital, she was markedly œdematous, her lower extremities being excessively so. She, however, spoke of no headache, nor of other unpleasant symptoms, excepting that arising from the mechanical distention of the legs and feet.

On July 18th, 1876, at 10 A. M., she was delivered by the interne, Dr. P. E. Loder. The position was a L. O. A. one. The child was a male, weighing six and a half pounds. The duration of the first and second stages of labor eight and a half hours. The placenta was delivered in twenty minutes. At the approach of night she became quite restless. She received twenty grains chloral hydrate, and slept well until five A.M. of the next day. At that time Dr. Loder was summoned. He found the patient recovering from a convulsion. This was not followed by coma. When quiet, after the seizure, her pulse was ninety per minute, and the temperature 100° Fahr. Two and a half hours later another convulsion appeared, quickly followed by another, and then convulsions occurred in rapid succession. At this time the pulse was 160 and the temperature $104\frac{1}{5}$° Fahr. Coma, becoming more and more profound, followed each paroxysm. The urine was now examined and found to be highly albumenous. Chloroform was administered and twelve dry cups were applied over the kidneys. At 10 A. M. her temperature was $105\frac{3}{5}$° Fahr., her pulse 168. The removal of the chloroform was quickly followed by a renewal of the convulsions. At 10:30 A. M. I reached the patient. Her temperature was still $105\frac{3}{5}^\circ$ Fahr., and her pulse 160, and the convulsions were found still to recur on withdrawal of the chloroform. From the coma she would merely pass into another convulsion.

I directed the continuance of the chloroform, and the administration of four drops of croton oil by the mouth, and later, had four drops administered by the rectum. Several free, watery, alvine evacuations followed, with a reduction of the temperature to 103° F., a fall of two and three-fifths degrees, but the pulse remained 160. The chloroform was now discontinued, and the convulsion did not return. The patient became almost perfectly conscious, conversed with those around, yet seemed in a state of bewilderment. Her thirst was intense, and she ate ravenously. Ice was also applied to her head. She now received thirty grains of potassium bromide and an ounce of whisky every two hours. At 10 P. M. her temperature was 103° F., and the pulse 160. She was restless and evidently failing. She now received one-quarter of a grain of sulphate of morphia hypodermically. At 4 A. M., of the 20th, after four hours of coma, she died. No autopsy obtained.

### CASE 5.—CONVULSIONS ON THE THIRTEENTH DAY AFTER LABOR—ALBUMENURIA—PERIMETRITIS—HIGH TEMPERATURE.

Loretta G., aged 23 years; a native of Pennsylvania; single; a primipara, and by occupation a domestic; was delivered on June 29th, 1876, at the Philadelphia Hospital, by Dr. Rush, then one of the internes. Nothing abnormal. The child was a male, weighing $7\frac{3}{4}$ pounds. The mother did very well until July 9th, when, after having been out of bed for a few hours, she was seized with a chill. The thermometer, after the chill, marked 104° F.; pulse 120. Eighteen grains of quinia were ordered to be taken during each twenty-four hours.

On the 10th she remained in bed and experienced no chill.

On the 11th, after again venturing out of bed, she was seized with a chill, followed by a temperature of 104° F., and a pulse of 120. There was no pain on pressure over the abdomen. There was slight headache.

On the 12th she felt well in the forenoon, but at 3 P.M. had another chill. At $6\frac{1}{2}$ P.M., of the same day, her temperature was $106\frac{2}{5}°$ F., and the pulse 150. At 7 P.M. the temperature was 107° F., pulse 180. At 7:30 P.M. convulsions appeared, it being the thirteenth day after labor. Immediately after the convulsion, the temperature was $103\frac{3}{5}°$ F., the pulse 186. She was now put into a wet pack. After remaining in this forty-five minutes, the temperature was reduced to 105° F., and the pulse to 150. But the convulsions repeated themselves. She was removed from the pack, and in twenty minutes the temperature had risen to 108° F. She was again put in the pack, and allowed to remain in it, without, however, any influence on the temperature.

I reached the patient at 1 A.M. I found her entirely unconscious and in a state of uninterrupted clonic, bilateral spasms, affecting, apparently, the entire voluntary muscular system. I directed the administration of coloroform. Urine, obtained by catheterization, showed a considerable amount of albumen. There was excessive tympanites. I directed an enema of oleum terebinthinæ and olive oil, but obtained no reduction of the tympanites. Five drops of croton oil, in olive oil, were now given by the mouth, but vomiting occurred. Five drops were now administered by the rectum, but this did not effect a fecal evacuation. The tympanites had now become so excessive, that a tube was passed into the large intestine, with, however, only a slight escape of gas. The chloroform, when freely given, held the convulsions in abeyance, but on even its partial removal the convulsions returned. The temperature was now 108° F., the pulse 140. The features were livid and puffy. Her condition seemed desperate, even hopeless. The croton oil would not act. I decided on venesection, as, under the circumstances, a desperate dernier resort. At 3 A.M. I took twenty-six ounces of blood from the arm, but without any perceptible influence on the convulsion, the chloroform being for the time suspended. There was no reduction in the temperature. The chloroform was again resorted to, and continued almost without intermission until the fatal result. Whisky and milk were also given at proper intervals.

At 11 A.M., July 13th, she died, having remained, almost until the last, in a state of clonic convulsions.

*Autopsy.*—Made forty-five minutes after death. Body well nourished and quite warm to the hand; lungs normal in appearance; old pleuritic adhesions, without, however, evidences of recent inflammation of either pleura. The pericardium contained two ounces of serum. The walls of the left ventricle were very decidedly thickened, hypertrophied; no lesions of the valves; both ventricles were empty and firmly contracted. The weight of the liver was four pounds nine ounces, and its tissue somewhat fatty. The right kidney weighed five and one half ounces, the left five ounces; both somewhat congested. A small, recent clot was found in the uterus. The uterine mucous membrane was normal in appearance, as also was the muscular tissue of the uterus. Pus and deposit of recent lymph were found on the peritoneal covering of the uterus. There was also a small amount of pus in the left ovary. There was decided congestion of the meningeal vessels. There was no hemorrhagic exudation, and no perceptible evidences of œdema of the brain tissue.*

Puerperal convulsions have, by different writers, been divided into several varieties, as hysterical, epileptic, apoplectic, and peculiar puerperal convulsions, to the latter the term eclampsia having been applied. High authority denies the practicability of this division. Cases 1 and 2 seem to me, however, to have been due to the pregnant state, and case 1 is an illustration of hysterical convulsions originating in the irritation of perhaps a naturally hyper-irritable nervous system, the irritation being reflected from the pregnant uterus. Case 2 seems to have been one of epilepsy, originating during labor, continuing through the subsequent non-pregnant condition, persisting through the next pregnancy, and appearing during the next delivery. I believe that epilepsy and hysteria may be thus associated with, or even dependent upon pregnancy; still, it seems to me that such convulsions must possess a more serious import than would be possessed by convulsions of a similar character occuring in the non-pregnant state, and, further, that they may merge into pure eclamptic seizures. Cases 3 and 4 are undoubtedly cases of puerperal convulsions

*Dr. P. E. Loder and other internes in the hospital deserve a great deal of credit for the faithful attention with which they devoted themselves to the care of the last two cases.

proper, while case 5 is so associated with complications, that though it may be included under the head of eclampsia, it is peculiar in its probable cause. The etiology of eclampsia is still undetermined. Modern pathology has modified the ideas of the profession. Cerebral congestion seemed at one time the accepted and always sufficient cause. It has not been thirty years since attention was first called to the association of albumenuria with pregnancy. Next the analogy between puerperal and uræmic convulsions was arrived at. But uræmia is not now recognized as explaining all cases, not even all cases of fatal convulsions.

It is not improbable that there are a number of morbid processes operating conjointly, sometimes one, sometimes another process becoming the most efficient cause. Let us, however, see in what respects the pregnant or puerperal individual differs from the same individual non-pregnant.

First, in reference to the circulatory fluid. Physiologists tell us that the modifications in this consist in a lowered specific gravity, an increased amount of water, an increase in the entire bulk of blood, an increase in white blood corpuscles, an increase in urea and in fibrin, a deficiency in the number of red blood corpuscles, and a deficiency in albumen; also, the disturbance in respiration permits the retention in the blood of more than the normal amount of carbonic acid. There is, in other words, a plethora, a fullness of the entire vascular system, but a plethora of hydræmic blood, with a deficiency in those elements destined for nutrition, and a superabundance of those elements representative of retrograde tissue metamorphosis, as urea and fibrin, and carbonic acid. Associated with blood alteration there is an increase in the propelling force of the heart; an increased vascular tension incident to a hypertrophied condition of the walls of the left ventricle, this hypertrophy being an accompaniment of probably every case of advanced pregnancy. This vascular tension is, of course, greatly increased during the powerful muscular efforts of delivery. Again, the nervous system undergoes a modification apart from its disturbed nutrition. This entire system is doubtless brought into more intimate relationships with the uterine system. Especially is this the case if, as Mr. Lee states, there are ganglia developed in the gravid uterus, ganglia which cannot be discovered in the non-pregnant womb. Let us add to these the mechanical interference with the abdominal circulation occasioned by the enlarged uterus. These various conditions are sufficient of themselves to occasion convulsions, especially if decided in degree, but more especially if more immediately determining causes are co-operating. The low specific gravity of the blood, its increased amount, and the increased force with which it is driven through the vessels, all co-operate in greatly increasing the process of exosmosis. This is evinced in the tendency to general anasarca. The intra-cranial tissues partake of this general condition, and there is at first an engorged state of their blood-vessels, then, by the increased exosmosis, an œdema of the brain tissue occurs. If this œdema is marked at the central portions, or the base of the brain, then convulsions may be occasioned, especially if to this state of œdema is added an irritable condition of the brain centre incident to perverted nutrition, and if, also, this increased intrinsic irritability is aggravated by irritation reflected from the peculiarly sensitive uterine nerves, or even if this reflected irritation originates in some part not included in the generative organs. Or it may be that emotional or atmospheric disturbances become the superadded cause.

But 90 per cent. of cases of puerperal convulsions are associated with albumenuria. The result is an aggravation of that condition of the system which exists in the pregnant woman without albumenuria. There is a further decrease in the highly nutritious element of the blood, albumen, a further lowering of the blood's specific gravity, a further increase in the already abnormal amount of urea. Uræmia becomes now the most active agent in the causation of the convulsions, but it is not acting alone; it is only the most prominent of the different etiological conditions; the albumenuria is itself, probably, but the result mainly of the disturbance in the relationships of the nutritive functions, a disturbance incident to the altered character of the circulatory fluid. Whether the increased amount of fibrin is directly or indirectly concerned in the production of the convulsions, has not, as far as I have been able to ascertain, been considered by writers on this subject. Yet, if we accept, as conclusive, the investigations of recent physiologists, fibrin, like urea, is a representative of broken-down tissue, and is an effete material, destined to be eliminated from the system. If effete, its presence in the blood in abnormal amounts must prove deleterious, and may probably be concerned, indirectly or directly, in the production of convulsions. Future pathology must determine this point for us.

In addition to the altered state of the blood that I have already referred to, must be included, as an additional cause, septicæmia and pyæmia.

Case 5 is one in which the repeated rigor, the excessive body heat before the convulsions, and the pelvic inflammation, would suggest as pyæmia, and that the pyæmia became the cause of the convulsive manifestations. It may have been the cause, either by the action of the poisoned blood in the nerve centres, or indirectly, by reason of excessive heating of the brain, as the thermometer marked 108° F. before the appearance of a convulsion. The presence of a moderate amount of albumen in the urine does not establish the presence of uræmia as the cause, since there were no symptoms of uræmia prior to the first seizure, and the urine was not tested until after the appearance of the convulsions. Albumenuria is occasionally not developed until after the convulsions have been in progress.

Cases 4 and 5 occurred also during the intense heat of the past summer, at a time when the thermometer was ranging in the neighborhood of 100° F. in the shade, and also but a short time before the terific electrical storm of July. Smellie, Simpson, and others have recognized the influence of excessive heat, and a high electrical state of the atmosphere, as causes determining convulsions.

The temperature in the two cases in which it was noted by the thermometer is of importance. In case 4 it was, after the first convulsion, 100° Fahr. As the convulsions recurred, it continued to rise, and attained a maximum of $105\frac{3}{5}$° Fahr., steadily rising during the continuance of the convulsions, and only fell when the convulsions ceased. It was not recorded during the coma that preceded death, and it is probable that it then rose above $105\frac{3}{5}$° Fahr. In case 5 the thermometer marked 108° Fahr. before the convulsions began, and ranged above 108° Fahr. until death, excepting when, for only a few minutes, it was reduced by the wet pack. In 1869 the temperature in puerperal convulsions was first noticed. In 1875 Bourneville published the first series of observations. Since then others have published observations, making in all 27 cases. In the seventeen cases reported by Bourneville, the thermometer never rose above 106° Fahr., and no case proved fatal in which the thermometer kept below 102° Fahr. This rise of the body temperature will probably be always found, in serious cases of eclampsia, influencing the prognosis and modifying the treatment. It seems strange that this important symptom should not have been noticed until within the last few years, the two cases that I have reported making, in all, but 29 published cases in which the temperature has been observed.

If we have properly understood the etiology and pathology of eclampsia, we can correctly arrive at the indications for treatment. *The prophylaxis should be closely attended to*, and should consist in the reduction of the blood mass, the elimination of the effete elements of the blood, and the renewal of its nutritive constituents.

To meet these three indications we must judiciously resort to venesection, diuretics and saline cathartics, and especially also to eutrophics. To these must be added the avoidance of all determining causes, emotional and, as far as possible, atmospheric, and especially the employment during delivery of such nerve and heart sedatives as chloroform, ether, chloral, etc. If convulsions appear before labor, the induction of labor must be determined by the severity of the seizures. If they occur during the first or second stages of labor, the earliest safe delivery is indicated, but it would be better to engage in no manipulation tending to the emptying of the uterus, unless the patient is fully etherized. Venesection after the incipiency of the convulsions, or even when the seizures were merely threatened, was before the time of anæsthesia invariably resorted to. To-day we must consider its indiscriminate employment as injudicious in the extreme; and yet its complete neglect is none the less unwarranted. Many cases, doubtless, demand its prompt employment, and we err if in such cases we neglect it. Very many cases now recover under the employment of anæsthesia without venesection. Chloroform, ether, chloral, opium, stand at the head of the narcotics.

Yet the resort to cathartics and diuretics is still indicated, though anæesthesia is resorted to. The intra-cranial vascular turgescence, the intra-cranial œdema, must be removed, the poisonous elements of the blood gotten rid of, at the same time that the system is brought under the quieting influences of the narcotic.

The state of the temperature will probably determine the beneficial or non-beneficial effects of our remedies, for it has been noticed that as the patient improves, there is a steady reduction in the temperature, and a steady rise if the case is progressing badly. We can thus ascertain her real condition, even when under complete anæsthesia; for if the narcotic is only smothering the convulsions, there will be but slight, if any, reduction in temperature, whereas if, while the patient is under anæsthesia, recovery is progressing, the thermometer will be the welcome harbinger. In this paper I can but deal in general statements, great as may be the temptation to analyze details.

One thing in reference to treatment, however, must be borne in mind, and that is, that we have no specific for this dreadful affection; that routine treatment will here prove as unsuccessful as it is unscientific; that neither in venesection nor in chloroform, nor in chloral, have we the only one thing needful. If this had been borne in mind in case 3, our patient's prospects would have been brightened.

In conclusion, Mr. President, allow me to ask the pardon of the Society for having so greatly trespassed on the limits recommended by the rules of the Society for similar papers. It would have been easier to have made the paper much longer; it was difficult to make it no longer than it is.—*Medical and Surgical Reporter.*

